# Heterologous Expression of Cryphonectria Hypovirus 1 in *Fusarium verticillioides* Reveals Stable Replication and Reduced Fumonisin Production

**DOI:** 10.3390/v18090971

**Published:** 2026-09-03

**Authors:** Sofía B. Ulla, Andrés G. Jacquat, María C. Cañizares, Martín G. Theumer, Ana I. López-Sesé, María D. García-Pedrajas, José S. Dambolena

**Affiliations:** 1 Facultad de Ciencias Exactas Físicas y Naturales (FCEFyN), Universidad Nacional de Córdoba (UNC), Córdoba 5000, Argentina; s.ulla@imbiv.unc.edu.ar; 2 Instituto Multidisciplinario de Biología Vegetal (IMBIV), Consejo Nacional de Investigaciones Científicas y Técnicas (CONICET), Avenida Vélez Sarsfield 1611, Córdoba X5016GCA, Argentina; 3 Unidad de Fitopatología y Modelización Agrícola (UFYMA), Consejo Nacional de Investigaciones Científicas y Técnicas (CONICET), Instituto Nacional de Tecnología Agropecuaria (INTA), Av. 11 de Sept No. 4755, Córdoba 5020, Argentina; andresgjacquat@gmail.com; 4 Instituto de Patología Vegetal, Centro de Investigaciones Agropecuarias, Instituto Nacional de Tecnología Agropecuaria (IPAVE-CIAP-INTA). Av. 11 de Sept No. 4755, Córdoba 5020, Argentina; 5 Instituto de Hortofruticultura Subtropical y Mediterránea “La Mayora”, Universidad de Málaga, Consejo Superior de Investigaciones Científicas (IHSM-UMA-CSIC), Estación Experimental “La Mayora”, Avenida Dr. Wienberg s/n, Algarrobo-Costa, 29750 Málaga, Spain; carmen.canizares@eelm.csic.es (M.C.C.); lopez-sese@eelm.csic.es (A.I.L.-S.); 6 Departamento de Bioquímica Clínica, Facultad de Ciencias Químicas (FCQ), Universidad Nacional de Córdoba (UNC), Córdoba 5000, Argentina; mgtheumer@unc.edu.ar; 7 Centro de Investigaciones en Bioquímica Clínica e Inmunología (CIBICI), Consejo Nacional de Investigaciones Científicas y Técnicas (CONICET), Haya de la Torre y Medina Allende, Ciudad Universitaria, Córdoba X5000HUA, Argentina

**Keywords:** *Fusarium verticillioides*, fungal viruses, heterologous host, mycotoxins, virocontrol

## Abstract

Mycoviruses represent promising tools for the biological control of phytopathogenic fungi. However, their application is often constrained by host specificity and low natural prevalence in certain pathogens. *Fusarium verticillioides*, a major maize pathogen and fumonisin B1 (FB1) producer, harbors very few known mycoviruses, a circumstance that limits the development of virus-based control strategies. In this study, we explored a virocontrol approach for *F. verticillioides* based on the use of a heterologous mycovirus, Cryphonectria hypovirus 1 (CHV1). For the artificial transfection of *F. verticillioides* with CHV1, we used an infectious cDNA clone of the virus and protoplast-mediated transformation. Through RT-PCR and double-stranded RNA (dsRNA) purification by chromatography on cellulose, it was confirmed that the CHV1 infectious cDNA clone is stably integrated into the fungal genome, giving rise to autonomous virus replication in the cytoplasm of *F. verticillioides* cells. Characterization of four independent transformants harboring CHV1 showed a significant reduction in FB1 production compared to the parental uninfected strain and only moderate alterations in vegetative growth. The CHV1-associated reduction in FB1 was confirmed in maize grown under both greenhouse and field conditions. Our findings provide evidence that heterologous mycoviruses can modulate mycotoxin production in *F. verticillioides,* thus expanding the pool of viral species that can be explored as potential biocontrol agents in this pathogen. Further work will be required to elucidate the underlying mechanisms of CHV1-associated downregulation of FB1 production in *F. verticillioides* and assess its potential application in virocontrol strategies.

## 1. Introduction

*Fusarium verticillioides* (Sacc.) Nirenberg (teleomorph: *Gibberella fujikuroi mating* population A) is a ubiquitous soil-borne fungus widely distributed in tropical and subtropical agroecosystems, where it persists as a saprophyte on crop residues and colonizes agricultural soils [1,2]. This species infects maize through multiple routes, including roots and silks, enabling systemic colonization as both an asymptomatic endophyte and, under certain conditions, a pathogenic organism that ultimately reaches developing kernels [3,4,5,6]. As the most frequently isolated fungus from maize kernels in both field and storage conditions, *F. verticillioides* is the primary causal agent of ear rot and contributes substantially to yield losses [7,8,9,10]. *Fusarium verticillioides* is also an active producer of mycotoxins that have a major impact on grain quality deterioration. Fumonisin B1 (FB1) from the B-series of the fumonisin family is the most relevant mycotoxin produced by *F. verticillioides* [7,11,12]. Once infection is established, fungicides have shown limited efficacy in reducing both disease severity and fumonisin accumulation [13,14]. Furthermore, there are increasing environmental and food safety concerns associated with the chemical control of plant diseases [15,16]. There is, therefore, a clear need to explore alternative, more effective, and sustainable strategies for the management of *F. verticillioides* and its mycotoxins, such as those based on the use of biological control agents.

Mycoviruses have attracted considerable attention due to their ability to modulate fungal physiology and plant–fungus interactions. In phytopathogenic fungi, they can cause hypovirulence (reduced virulence) and are primarily studied as potential targeted biological control agents [17]. Mycoviruses with double-stranded RNA (dsRNA) or positive-sense single-stranded RNA (+ssRNA) genomes, which may be either encapsidated or exist as capsidless RNA replicons, are considered the prevalent groups [18]. However, the application of next-generation RNA sequencing approaches to the discovery of novel mycoviruses is providing evidence that their genome organizations are more complex and diverse than previously thought [19,20]. Unlike many plant and animal viruses, mycoviruses generally lack an extracellular phase in their life cycles. Their transmission depends on fungal biology, occurring horizontally via hyphal anastomosis (fusion of vegetative hyphae) and vertically through sexual and asexual spores. This transmission only by intracellular routes, often restricts their spread to genetically compatible strains of the same species [17,18].

The search for mycoviruses with potential in biological control is generally focused on identifying those naturally infecting the target pathogen and characterizing their effect on virulence. In *F. verticillioides,* though, the incidence of natural mycovirus infections appears to be very low. A survey of 99 *F. verticillioides* isolates led to the identification of a single mycovirus, Fusarium verticillioides mitovirus 1 (FVM1) [21], and no other mycoviruses from this species have been reported in the literature. Hence, alternative strategies may be required to harness mycoviruses for biological control in this pathosystem. These can include the use of heterologous viruses, involving the artificial introduction of mycoviruses known to confer hypovirulence in one fungal species into a different target host. Experimental studies have demonstrated successful cross-species transmission of hypovirulence-associated mycoviruses, with retention of key phenotypic effects in the new host, thus supporting the feasibility of this strategy in the absence of suitable native viruses. Cryphonectria hypovirus 1 (CHV1; family *Hypoviridae*), infecting the chestnut blight fungus *Cryphonectria parasitica*, represents the best-characterized example of a hypovirulence-inducing mycovirus and has been widely used as a model for studying virus–fungus interactions [18,22,23]. This has prompted the development of artificial transfection methods that can be taken advantage of for the transfection of non-host fungal species. Initial studies focused on the transmission of CHV1 to fungal species phylogenetically related to its natural host. In *C. parasitica*, CHV1 infection induces marked phenotypic alterations, including reduced virulence, altered pigmentation, and changes in growth and sporulation. Many of these phenotypic alterations were also observed in other members of the order *Diaporthales* infected with CHV1 [24,25,26]. More recent works have shown that CHV1 also has the ability to replicate in fungal species phylogenetically distant from *C. parasitica*, such as *Fusarium graminearum* and *Fusarium oxysporum,* inducing phenotypic alterations [27,28]. However, whether CHV1 can replicate in *F. verticillioides* remains unknown. Nonetheless, a recent study by Ma et al. [29] reported that the ourmia-like virus FsoOLV1, originally identified in *Fusarium solani*, can be experimentally introduced into *F. verticillioides* by protoplast transfection, where it is stably maintained and associated with reduced virulence, providing initial evidence that heterologous mycovirus introduction is feasible in this species.

In this context, the objective of the current study was to ascertain the ability of CHV1 to independently replicate in *F. verticillioides* upon artificial transfection with a cDNA infectious clone, and to assess the resulting virus-associated phenotypic effects in this heterologous host. We demonstrated that transformation of *F. verticillioides* with a construct containing a cDNA copy of the CHV1 genome under the regulation of a fungal promoter gives rise to autonomous viral replication in this non-native host. Furthermore, we showed that CHV1 infection is associated with phenotypic alterations in *F. verticillioides*, most notably a significant reduction in FB1 production in maize. Our results therefore reveal the potential of using heterologous mycoviruses to control mycotoxin production in this pathogen.

## 2. Materials and Methods

### 2.1. Fungal Strains and General Growth Conditions

*Fusarium verticillioides* M3125 (FGSC #7600), kindly provided by Dr. Robert Proctor (USDA, Agricultural Research Service, National Center for Agricultural Utilization Research, Peoria, IL, USA), was used as the wild-type (WT) parental strain for all transformations. Potato dextrose agar (PDA) and potato dextrose broth (PDB) were used as media for solid and liquid cultures, respectively. Selective media for transformed strains consisted of PDA supplemented with 150 μg/mL hygromycin (Hyg) or 200 μg/mL nourseothricin (NTC). Czapek Dox Agar (CDA) (OXOID, Basingstoke, Hants, UK) was used for assays of horizontal virus transmission. Plates were incubated at 25 °C in the dark and liquid cultures at 28 °C with shaking at 200 rpm.

### 2.2. Transformation of Fusarium verticillioides with the CHV1 Infectious Clone

*Fusarium verticillioides* was transformed with the CHV1 cDNA infectious clone pXH9 (kindly provided by Prof. N. Suzuki) via protoplast-mediated transformation. Protoplasts were prepared from germinated conidia following the protocol of Lopes et al. [30] with modifications. Conidia were germinated by inoculating 1 × 10^8^ freshly obtained conidia in 100 mL of PDB and incubating them for 11 h at 28 °C with shaking at 200 rpm. Then they were collected by filtration through a Miracloth membrane (Calbiochem Miracloth, EMD Millipore, Billerica, MA, USA), washed with 45 mL of OM buffer (1.2 M MgSO_4_; 10 mM Na_2_HPO_4_), and finally resuspended in 20 mL of OM buffer containing 50 mg Driselase (Sigma-Aldrich, St. Louis, MO, USA) and 1 g Extralyse (LAFFORT^®^, Bordeaux, France). Digestion of cell walls was carried out by incubating samples at 28 °C and 50 rpm for 2–3 h. After that, protoplasts were collected by filtration through 40 μm nylon filters, washing with ice-cold STC buffer (10 mM Tris-HCl, pH 7.5; 50 mM CaCl_2_; 0.8 M sorbitol). The filtrate was centrifuged in a swing-bucket rotor for 15 min at 3000 rpm and 4 °C, and the pelleted protoplasts were resuspended in 400 μL STC buffer and counted in a Fuchs–Rosenthal hemocytometer. Finally, protoplast suspensions were adjusted to a concentration of 3 × 10^8^ protoplasts/mL and directly used for transformation or stored at −80 °C in 100 µL aliquots. For storage at −80 °C, dimethyl sulfoxide (DMSO; Sigma-Aldrich, St. Louis, MO, USA) at a final concentration of 7% was added.

Protoplasts thus prepared were transfected with the virus infectious clone using a polyethylene glycol-mediated transformation protocol. Infectious clone pXH9 carries a full-length cDNA copy of the CHV1 strain EP713 genome (NCBI GenBank: M57938.1) under the regulation of the *C. parasitica* constitutive glyceraldehyde-3-phosphate dehydrogenase promoter, and a *hygR* cassette for the selection of transformants [31]. Transformation of *F. verticillioides* with pXH9 was conducted as described by Cañizares et al. [28] with modifications. Briefly, 100 μL aliquots of the protoplast suspension (3 × 10^7^ protoplasts) were transformed with 10 μg of pXH9 DNA. To generate free DNA ends to promote integration of the infectious clone into the genome of *F. verticillioides* upon transformation, pXH9 was partially digested with restriction enzyme *Xba*I (Figure S1). For the partial digestion, 30 µg of pXH9 DNA was incubated with 10 units of *Xba*I (Takara Bio Inc., Kusatsu, Shiga, Japan) for 10 min at 37 °C, and then the enzyme was heat-inactivated at 65 °C for 20 min. An aliquot of the digestion mixture was analyzed by gel electrophoresis prior to transformation to confirm that, under these conditions, most of the DNA corresponded to partially digested linearized plasmid (Figure S1). Protoplasts and the partially digested pXH9 vector DNA were incubated on ice for 30 min. Then, 1.0 mL of PTC (10 mM Tris-HCl, pH 7.5; 100 mM CaCl_2_; 0.8 M sorbitol; 40% PEG 4000) was added, and samples were incubated at room temperature for 25 min. After transformation, protoplasts were recovered by centrifugation at 4000× *g* for 5 min, resuspended in 600 μL of STC, and spread onto regeneration minimal medium plates (RMM: sucrose 200 g/L, glucose 20 g/L, NaNO_3_ 2 g/L, KH_2_PO_4_ 1 g/L, MgSO_4_·7H_2_O 0.5 g/L, KCl 0.5 g/L, agar 12.5 g/L). Plates were incubated for 16 h at 28 °C in the dark to promote regeneration of cell walls, and then selection was applied by overlaying each plate with 5 mL of top agar medium (RMM but with only 5 g/L agar) containing 90 µL of hygromycin B (50 mg/mL). When hygromycin-resistant (*hygR*) colonies emerged, they were individually transferred to PDA supplemented with 150 μg/mL of hygromycin to confirm stable integration of pXH9 carrying the hygromycin B phosphotransferase (*hph* gene) expression cassette.

### 2.3. Molecular Analysis of Transformants

Integration of pXH9 in the genome of transformed *hygR* colonies was analyzed by PCR using CHV1-specific primers. As mentioned above, the pXH9 DNA used for transformation was partially digested with *Xba*I, which contains two recognition sites in the infectious clone. pXH9 was partially digested so that most molecules used in the transformation harbored the entire vector but in a linearized form to promote its integration into the fungal genome. One of the *Xba*I sites of pXH9 is located at the 5′ end of the *hph* cassette, whereas the other one interrupts the ORF-B of CHV1. Therefore, the initial analysis of transformants included PCR with primer pair CHV1-F2 and CHV1-R3 (Figure S1; Table S1) that allow verifying in which transformants had pXH9 integrated without disrupting the CHV1 ORF-B. Transformants with correct integration of the infectious clone were then subjected to RT-PCR to corroborate expression of the CHV1 coding RNA from the fungal promoter. For RT-PCR, total RNA was extracted using the Spectrum Plant Total RNA kit (Sigma-Aldrich, St. Louis, MO, USA) and treated with ezDNase^TM^ (Invitrogen, Carlsbad, CA, USA). RT reactions were performed with primer CHV1 R1 (Table S1) using SuperScript III reverse transcriptase (Invitrogen, Carlsbad, CA, USA), following the manufacturer’s instructions. Then, 5 µL of each cDNA sample was used as a template for PCR amplification with primer combination CHV1 F1 and CHV1 R1. PCR reactions were carried out in a total volume of 50 μL using BIOTAQ™ DNA Polymerase (Meridian Bioscience, Cincinnati, OH, USA) according to the manufacturer’s instructions.

To confirm the presence of the CHV1 dsRNA indicative of independent replication of the virus in the cytoplasm of *F. verticillioides* cells, transformants shown to be expressing CHV1 cDNA according to the RT-PCR results were subjected to chromatography on cellulose as described by Valverde et al. [32]. Chromatography on cellulose extracts was prepared from 5 g of fungal material reduced to a fine powder in a mortar and pestle in the presence of liquid nitrogen, treated with DNase I (Roche, Basel, Switzerland) to eliminate residual genomic DNA, and resolved on 0.8% agarose gels for visualization of the dsRNAs.

Accumulation levels of CHV1 in the different transformants harboring the virus were assayed by RT-quantitative PCR (RT-qPCR). For that, total RNA extraction and RT reactions were performed, as described above, for regular RT-PCR. qPCR reactions were then prepared in a 10 µL final volume containing SensiFAST™ SYBR No-Rox Kit (Meridian Bioscience, Cincinnati, OH, USA) master mix, the virus-specific primers CHV1 F1 and CHV1 R1, and 2 µL of a dilution 1/10 of the cDNA sample. Three technical replicates were included for each of the three biological replicates, and PCR-grade water was used instead of DNA as a no-template control. The amplification was carried out in a CFX96 Touch Real-Time PCR System (Bio-Rad Laboratories, Hercules, CA, USA). The thermal cycling protocol started at 94 °C for 2 min and followed 35 cycles of 94 °C for 30 s, 60 °C for 30 s (signal acquisition), 72 °C for 30 s, and a final melting curve. Relative viral quantification levels from three biological replicates and three technical replicates were fold-determined using the 2^−ΔΔ^*^C^*^q^ method [33], and statistical differences were analyzed with the software IBM SPSS Statistics (v32). The fungal *actin* gene, amplified using primers act q7 and act q8 [34], was used to normalize virus RNA levels.

### 2.4. Analysis of the Effect of CHV1 on Fusarium verticillioides Vegetative Growth and Fumonisin Production

To assay how CHV1 affects vegetative growth and conidia production in *F. verticillioides*, PDA plates were inoculated at the center with 5 μL of a conidial suspension (1.0 × 10^6^ conidia/mL) of virus-free and infected strains. Plates, five replicates per strain, were then incubated at 25 °C until colonies covered the entire plate surface. During this period, colony diameters were measured daily to calculate growth rates. At the end of the growth period (6–7 days), conidia were harvested by washing plates with 0.5% Tween-80 and filtering the suspension through a Miracloth membrane. Conidial concentrations were determined using a Fuchs–Rosenthal hemocytometer.

FB1 production was evaluated on maize kernels inoculated with the different *F. verticillioides* strains following the method described by Brito et al. [35]. Before inoculation, maize kernels were placed in 250 mL Erlenmeyer flasks and sterilized by autoclaving on two consecutive days for 15 min at 121 °C. Water was added to reach 35% moisture. Maize was inoculated with 1 mL of a conidial suspension (1.0 × 10^6^ conidia/mL) of each fungal strain and incubated in the dark at 25 °C for 28 days; uninoculated flasks served as controls. Toxin extraction and purification from fermented maize followed a modified Voss et al. [36] protocol. A dried corn sample (10 g) was ground, and FB1 was extracted with ultrapure water by orbital shaking for 2 h. Extracts were centrifuged at 9000× *g*, and the supernatant was recovered. A 500 μL aliquot of the supernatant was mixed with 500 μL acetonitrile prior to analysis. FB1 quantification was performed by HPLC with fluorescence detection according to Shepard et al. [37]. The FB1 concentrations were determined by comparison with calibration curves generated from commercial FB1 standards (PROMEC, Tygerberg, South Africa). Six replicates were prepared per strain, and the experiment was repeated twice.

### 2.5. Analysis of CHV1 Transmission in Fusarium verticillioides

Horizontal transmission of CHV1 in *F. verticillioides* via hyphal anastomosis was assessed by pairing virus donor and recipient strains on CDA plates. *Fusarium verticillioides* transformant XH9-6 and a nourseothricin sulfate (Jena Bioscience, Jena, Germany) (NTC)-resistant (NTC^r^) virus-free strain [38] were used as virus donor and recipient strains, respectively. CDA plates were co-inoculated with mycelial plugs of these two strains, placing them 1–1.5 cm apart, and then incubated at 25 °C in the dark to allow strains to grow and come into contact. At 30 days post-inoculation, fungal material was taken from different points of the area of contact of both strains and transferred to PDA supplemented with NTC to select recipient isolates. The presence of CHV1 in recipient NTC^r^ colonies was assessed by RT-PCR using CHV1-specific primers, as previously described for the molecular analysis of transformants.

Transmission of CHV1 to conidia to confirm viral infection stability through asexual reproduction was evaluated by generating monoconidial cultures from the *F. verticillioides* transformants confirmed to be infected with CHV1. Single-spore isolates were cultured in PDB and in the dark at 25 °C for 7 days. Mycelia were harvested by filtration, and total RNA was extracted using the Spectrum™ Plant Total RNA Kit (Sigma-Aldrich, St. Louis, MO, USA). Residual genomic DNA was removed by DNase I (Thermo Fisher Scientific, Waltham, MA, USA) treatment. The presence of CHV1 was then assessed by subjecting RNAs to RT-PCR using CHV1-specific primers, as described above.

### 2.6. Phytopathogenicity Assays

Symptom severity assays under greenhouse and field conditions were conducted on a commercial maize hybrid susceptible to Fusarium ear rot to evaluate the potential hypovirulent effect of CHV1 in the heterologous host *F. verticillioides*. Under semi-controlled greenhouse conditions, superficially sterilized maize seeds were sown in individual pots. Under field conditions, seeds were manually sown in linear rows at 20 cm spacing. Irrigation was applied manually in both settings throughout the experiment.

Ears were inoculated by injecting 2 mL conidial suspension (1.0 × 10^6^ conidia/ mL) into the silk canal using an automatic syringe, approximately 6 days after silk emergence, following the protocol of Iglesias et al. [10]. Inoculated ears were covered with kraft paper envelopes until harvest to limit isolate spread and environmental contamination by *Fusarium* spp. Treatments included conidial suspensions of transformed *F. verticillioides* strain XH9-6 confirmed to be infected with CHV1, the parental WT uninfected strain, and sterile water as a negative control. Assays were terminated at plant senescence and ear maturity.

In the greenhouse assay, FB1 content was determined in five individual ears per treatment (*n* = 5 per treatment). In the field assay, symptom severity was assessed on each of the ears harvested per treatment (*n* = 20 per treatment) using a modified Enerson and Hunter [39] scale. For chemical analyses, all harvested ears per treatment were ground and pooled into a single composite sample per treatment to simulate standard agronomic sampling procedures and obtain a representative lot value. It was subsequently divided into five analytical sub-samples for the quantification of FB1 and ergosterol content. FB1 content in harvested grains was determined, as described in Section 2.4. Ergosterol quantification followed a modified protocol based on Young [40].

### 2.7. Statistical Analyses

Statistical analyses of growth rate, conidia production, and fumonisin B1 production data were performed by one-way analysis of variance (ANOVA) (*p* ≤ 0.05). The normality and homogeneity of variance were tested. Data are presented as mean ± standard error, and differences between means were considered significant if *p* ≤ 0.05. Di Rienzo, Guzmán, and Casanoves test (DGC) was used for mean comparisons.

## 3. Results and Discussion

### 3.1. Artificial Transfection of Fusarium verticillioides with a Cryphonectria Hypovirus 1 (CHV1) cDNA Infectious Clone Results in Autonomous Viral Replication

Traditionally, research on mycoviruses as potential biological control agents has focused on the identification and characterization of those that naturally infect target fungal species. In *F. verticillioides,* despite its significant health and agronomic impact, mycovirus discovery efforts have yielded scarce results; to date, only a single virus, Fusarium verticillioides Mitovirus 1 (family *Mitoviridae*), has been described in this species [21]. In this context, the use of heterologous mycoviruses emerges as a promising alternative strategy, potentially accelerating the development of mycovirus-based control approaches by overcoming the limited availability of naturally occurring viral infections in this fungus.

The use of heterologous mycoviruses, as addressed in the present study, involves several key challenges. First of all, as mycoviruses are predominantly transmitted by intracellular mechanisms, the infection of a non-host species generally requires the availability of artificial transfection methods. Secondly, the heterologous mycovirus has to have the ability to efficiently replicate in the new host so that, once artificially introduced, the infection is stably maintained. In this study, we tested transformation with a CHV1 cDNA infectious clone, pXH9, as the approach to artificially infect *F. verticillioides* with this hypovirus. The vector pXH9, initially developed for the transfection of the CHV1 natural host, contains a full-length cDNA copy of the CHV1 genome flanked by the *C. parasitica* Pgpd and Tgpd sequences, and a *hygR* marker for the selection of transformants (Figure S1) [31]. The initial studies involving *C. parasitica* confirmed that upon introduction of pXH9 via protoplast-mediated transformation and its integration in the fungal genome, the CHV1 cDNA is transcribed into RNA under the control of the constitutive Pgdp promoter, generating viral transcripts that serve as template for the synthesis of the RNA-dependent RNA polymerase (RdRp), which, in turn, initiates the replication cycle of the virus in the host cell cytoplasm [31,41,42]. Later studies showed that pXH9 behaves similarly in various non-host fungal species of the Ascomycota, successfully promoting autonomous replication of CHV1, and that this infectious clone can therefore be directly used for the infection of fungal species other than *C. parasitica* [26,28].

**Figure 1:**
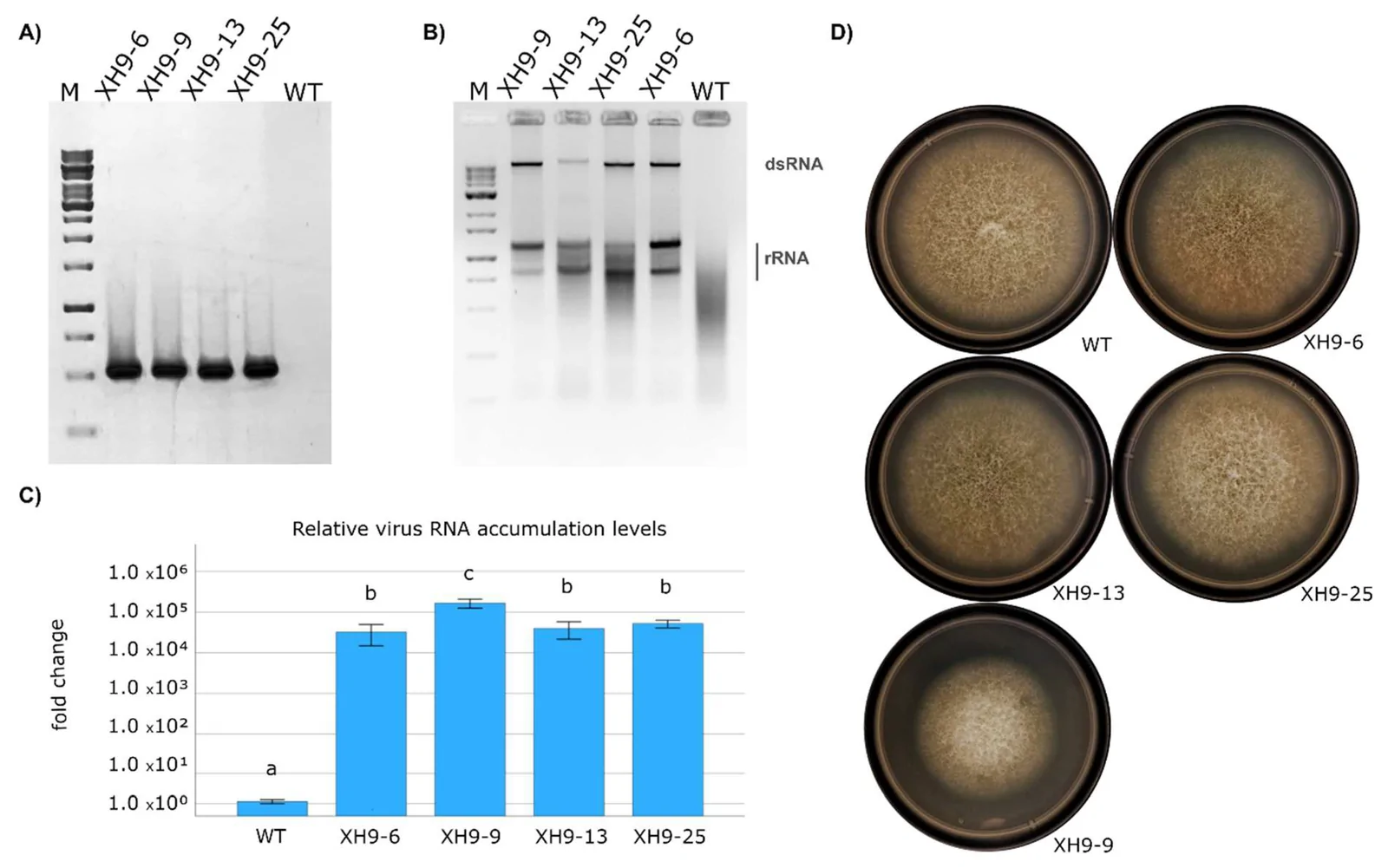
Confirmation of Cryphonectria hypovirus 1 replication in the heterologous host *Fusarium verticillioides.* In an attempt to infect *F. verticillioides* with Cryphonectria hypovirus 1 (CHV1), fungal protoplasts were transformed with the cDNA infectious clone pXH9 [41]. (**A**) Confirmation of CHV1 coding RNA transcription upon integration of pXH9 in the fungal genome; four independent transformants, designated XH9-6, XH9-9, XH9-13, and XH9-25, were subjected to RT-PCR with CHV1-specific primers. (**B**) Confirmation of CHV1-independent replication in the four selected transformants through detection of the virus double-stranded RNA (dsRNA) intermediate of replication by chromatography on cellulose. RT-PCR products and chromatography on cellulose extracts were analyzed by agarose gel electrophoresis. Lanes: M, 1 kb DNA Ladder (Thermo Scientific); WT, untransformed WT strain. rRNA, residual ribosomal RNA. (**C**) Analysis of CHV1 accumulation levels by quantitative RT-PCR. Relative virus RNA accumulation levels (fold change) were estimated by using 2^−ΔΔCq^ values from mean cycle threshold values normalized to the fungal actin gene expression levels. Statistical analyses were performed on ΔCq values. Bars represent standard errors calculated from three biological replicates. Values with the same letters are not significantly different according to Tukey tests (*p* ≤ 0.05 level). (**D**) Representative top-view photographs of WT and CHV1-infected strains grown on PDA for 7 days in the dark at 25 °C.

To generate free DNA ends to promote integration of pXH9 in the genome of *F. verticillioides* upon protoplast-mediated transformation, we linearized it by partial digestion with the restriction enzyme *Xba*I. There are two *Xba*I sites in pXH9, and one of them interrupts the ORF-B of CHV1. Hence, the initial molecular analysis of transformants involved PCR with CHV1-specific primers CHV1-F2/CHV1-R3 (Table S1), which generate a 3980 bp amplicon only when pXH9 integrates without disrupting the virus ORF-B (Figure S1). Four independent transformants with a positive PCR result indicative of correct integration of pXH9 in the *F. verticillioides* genome were selected for further analysis; they were designated strains XH9-6, XH9-9, XH9-13, and XH9-25. Efficient transcription from the *C. parasitica* Pgdp promoter is a requirement to attain independent replication of CHV1 in *F. verticillioides* upon transformation with pXH9. An RT-PCR analysis of strains XH9-6, XH9-9, XH9-13, and XH9-25 using CHV1-specific primers confirmed the presence of the virus transcript in all four strains, whereas no amplification was observed in the untransformed parental *F. verticillioides* strain (Figure 1A). To evaluate the stability of the infectious clone construct integration in the *F. verticillioides* genome and expression of the virus RNA, we then tested for the presence of CHV1-derived transcripts in monosporic subcultures obtained from strains XH9-6, XH9-9, XH9-13 and XH9-25. Ten single-spore cultures from each strain were tested by RT-PCR, and all of them showed CHV1 transcription. This RT-PCR analysis was repeated across three successive generations, confirming that single-spore cultures consistently gave a positive result for the presence of CHV1 transcripts.

To corroborate independent replication of CHV1 in the cytoplasm of *F. verticillioides* cells of strains XH9-6, XH9-9, XH9-13, and XH9-25, we then tested for the presence of the virus dsRNA intermediate of replication, using chromatography on cellulose. Detection of large dsRNA molecules by cellulose-affinity purification followed by DNase and S1 nuclease treatment remains a reliable approach for distinguishing viral dsRNA from host nucleic acids [28,43,44]. The electrophoretic analysis of the chromatography on cellulose extracts prepared from the four independent transformants with a positive RT-PCR result showed the presence of a dsRNA band corresponding to the full size of the CHV1 genome in all of them, whereas no dsRNA band was detected in the wild-type uninfected control (Figure 1B). This result is consistent with active independent CHV1 replication in *F. verticillioides*. Thus, we corroborated the ability of CHV1 to infect *F. verticillioides* replicating independently, and proved that the *C. parasitica* infectious clone works successfully in initiating the replication cycle in this *Fusarium* host. This finding is consistent with previous reports demonstrating CHV1 replication in other *Fusarium* species [28,45]. The four *F. verticillioides* transformed strains exhibiting independent replication of CHV1 were also analyzed by RT-qPCR to determine accumulation levels of the hypovirus. Strains XH9-6, XH9-13, and XH9-25 showed similar viral accumulation levels. XH9-9, on the other hand, exhibited a small but statistically significant increase in relative virus accumulation levels as compared with the other three strains (Figure 1C). The higher viral accumulation observed in transformant XH9-9 may arise from several factors, including genomic insertion-site effects and potential multiple cDNA integrations. Although the use of a linearized plasmid generally reduces the likelihood of multiple insertions, the possibility of multiple cDNA integrations cannot be completely excluded.

### 3.2. CHV1 Infection Does Not Have a Marked Impact on Fusarium verticillioides Vegetative Growth

To gain initial insight into the contribution of this heterologous mycovirus to fungal biology, we evaluated key phenotypic traits associated with dispersal, substrate colonization, and pathogenic potential of *F. verticillioides* infected with CHV1. We started by quantifying vegetative reproduction (conidiation) and radial growth rates as proxies for an initial assessment of how CHV1 would affect fungal fitness and capacity to colonize maize.

Strains XH9-6, XH9-9, XH9-13, and XH9-25 all exhibited a reduction in growth rate statistically significant compared to the WT uninfected strain (Table 1). However, there were also differences in growth rate among them. The most pronounced reduction was observed in XH9-9 (~50.9%), followed by XH9-6 (~21.5%), whereas XH9-13 and XH9-25 displayed growth rates closer to the parental control (~9.1% and ~9.9% reduction, respectively). This result provides evidence that CHV1 affects growth rates in *F. verticillioides*, as all strains harboring the virus exhibited reduced growth, although to a different extent. Interestingly, the strain with the most marked reduction in growth rate, XH9-9, was also the one with the highest levels of virus accumulation according to the RT-qPCR analysis (Figure 1C). A potential explanation of this result is that the effect of CHV1 on growth rate is dose-dependent; hence, higher virus titers induced a more pronounced reduction in growth. However, it cannot be ruled out that other factors in addition to CHV1 infection are contributing to the clearly reduced growth of this strain. The conidiation analysis revealed that only strain XH9-13 exhibited a significant reduction in conidia production (~76%) compared to the WT strain (Table 1). As the effect on conidiation was observed in only one of the four strains confirmed to exhibit replication of CHV1, it does not appear to be associated with the virus. No apparent differences in macroscopic morphology were observed among the strains under the conditions evaluated (Figure 1D). The mycovirus CHV1 is well known for inducing marked phenotypic alterations in its natural host, *C. parasitica*, including reduced vegetative growth, altered pigmentation, decreased conidiation, and pronounced hypovirulence [23,46,47]. Effects of CHV1 on growth, morphology, conidiation, and pathogenicity have also been reported in heterologous hosts, although the magnitude and direction of these effects may vary among species [24,25,28]. In comparison with other heterologous hosts, the effect of CHV1 on altering *F. verticillioides* phenotypic growth traits was moderate.

### 3.3. In Fusarium verticillioides CHV1 Infection Leads to a Significant Reduction in FB1 Production but Has a Limited Effect on Disease Severity

Production of the mycotoxin FB1 by *F. verticillioides* is a major concern. Bearing this in mind, one of the key aims of the present study was to investigate whether CHV1 affects FB1 production in this species. To this end, we first quantified FB1 production by strains XH9-6, XH9-9, XH9-13, and XH9-25, as well as the virus-free WT strain, grown on previously autoclave-sterilized maize kernels. We observed a consistent effect of CHV1 in reducing FB1 production; all strains harboring CHV1 produced significantly less FB1 than the WT, with no significant differences among them (Table 2). Specifically, FB1 levels in maize kernels decreased by approximately 34% to 71.6% when inoculated with the four strains where replication of CHV1 had been confirmed.

**Figure 2:**
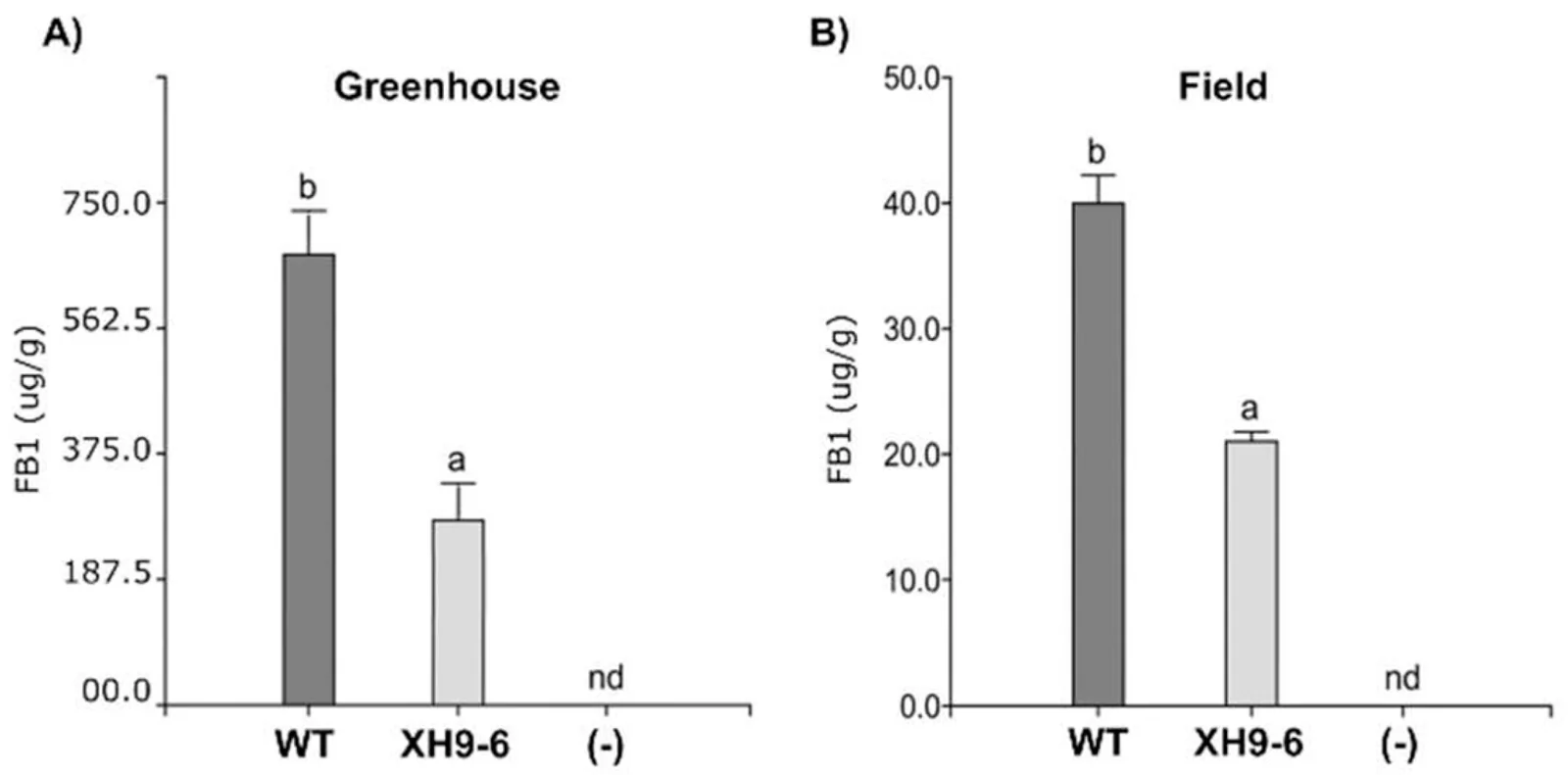
Effect of CHV1 on fumonisin B1 content in ears of maize grown under greenhouse and field conditions. The silks were inoculated with conidial suspensions of XH9-6 and the virus-free parental (WT) strain. (**A**) Greenhouse assay: Maize plants were grown in pots (*n* = 5 per treatment), and FB1 content was evaluated in five individual ears per treatment. (**B**) Field assay: Plants were grown directly in the soil under field-like conditions. The samples from all harvested ears per treatment (*n* = 20 per treatment) were pooled to obtain a composite sample, which was analyzed in five subsamples. FB1 content is expressed as µg of FB1 per gram of dry maize (µg/g dry weight). Bars with different letters indicate significant differences in fumonisin B1 content, according to the DGC multiple comparisons test (*p* < 0.05). nd: means not detected.

To examine in more detail the CHV1-associated reduction in FB1 production in *F. verticillioides*, we then tested if this effect persists in intact plants. For this purpose, we conducted experiments under two conditions: semi-controlled greenhouse settings and field-like, non-controlled environments. For these experiments, we selected one of the CHV1-infected strains, XH9-6, considering that they are technically more complex and that all CHV1-infected strains had shown a similar behavior regarding FB1 production in maize kernels (Table 2). Strain XH9-6 exhibited a representative phenotype among the evaluated transformants, including a clear reduction in FB1 production without the more extreme alterations observed in other strains (e.g., the pronounced growth defect of XH9-9 or the reduced conidiation of XH9-13). As such, XH9-6 provides a balanced and stable experimental model for assessing virus-associated effects under more complex biological conditions, minimizing potential biases arising from strain-specific phenotypes not directly attributable to CHV1 activity. In both greenhouse and field assays, ears inoculated with XH9-6 exhibited significantly lower FB1 levels compared to those infected with the WT. The FB1 accumulation in ears inoculated with XH9-6 reached approximately half of the levels observed in WT-infected maize, while remaining undetectable in the water control (Figure 2).

**Figure 3:**
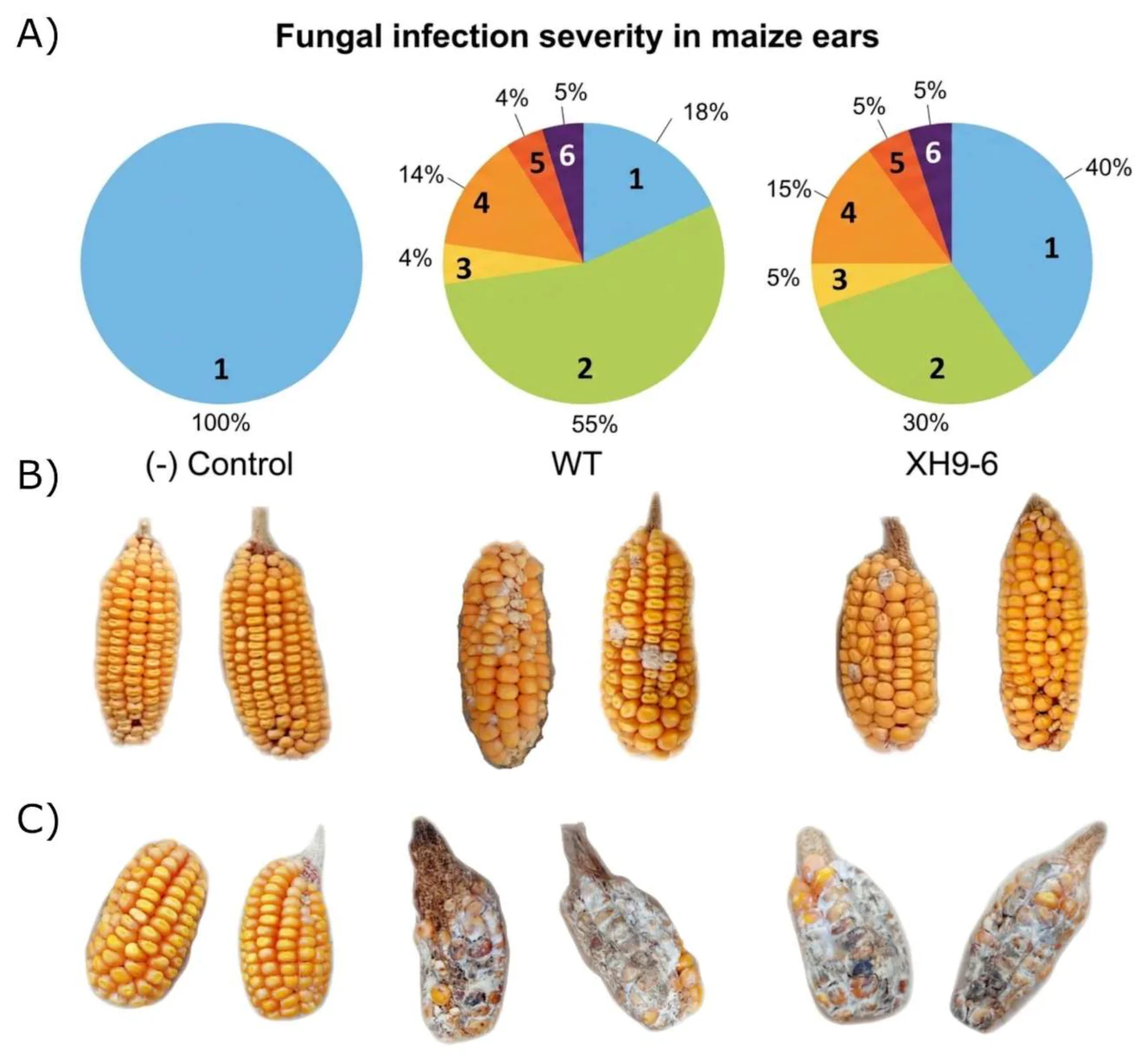
Disease severity in maize ears. (**A**) Disease severity in maize ears grown under field-like conditions. To assess the damage caused by fungal infection, a modified Enerson and Hunter [39] scale was used. The scale comprises seven severity classes (numbers within the graphs), each corresponding to a percentage range of affected ear area: grade 1, no visible damage (0%); grade 2, 1–3%; grade 3, 4–10%; grade 4, 11–25%; grade 5, 26–50%; grade 6, 51–75%; and grade 7, 76–100% (not observed in this study). Percentages shown outside the graphs indicate the proportion of ears in each treatment corresponding to each severity grade. *n* = 20. (**B**) Representative images of maize ears from the field-like experiment. (**C**) Representative images of maize ears from the greenhouse experiment.

Nevertheless, disease severity assessments under greenhouse conditions showed that both WT and XH9-6 induced severe symptoms with no significant differences between treatments (Figure 3C). In field-like conditions, while both strains caused detectable disease, plants inoculated with XH9-6 consistently exhibited a modest reduction in symptom severity relative to the WT (Figure 3A,B). The absence of differences under greenhouse conditions may be related to the fungal load used for artificial inoculation, which may have been disproportionately high relative to the ear size of maize plants grown individually in pots.

**Figure 4:**
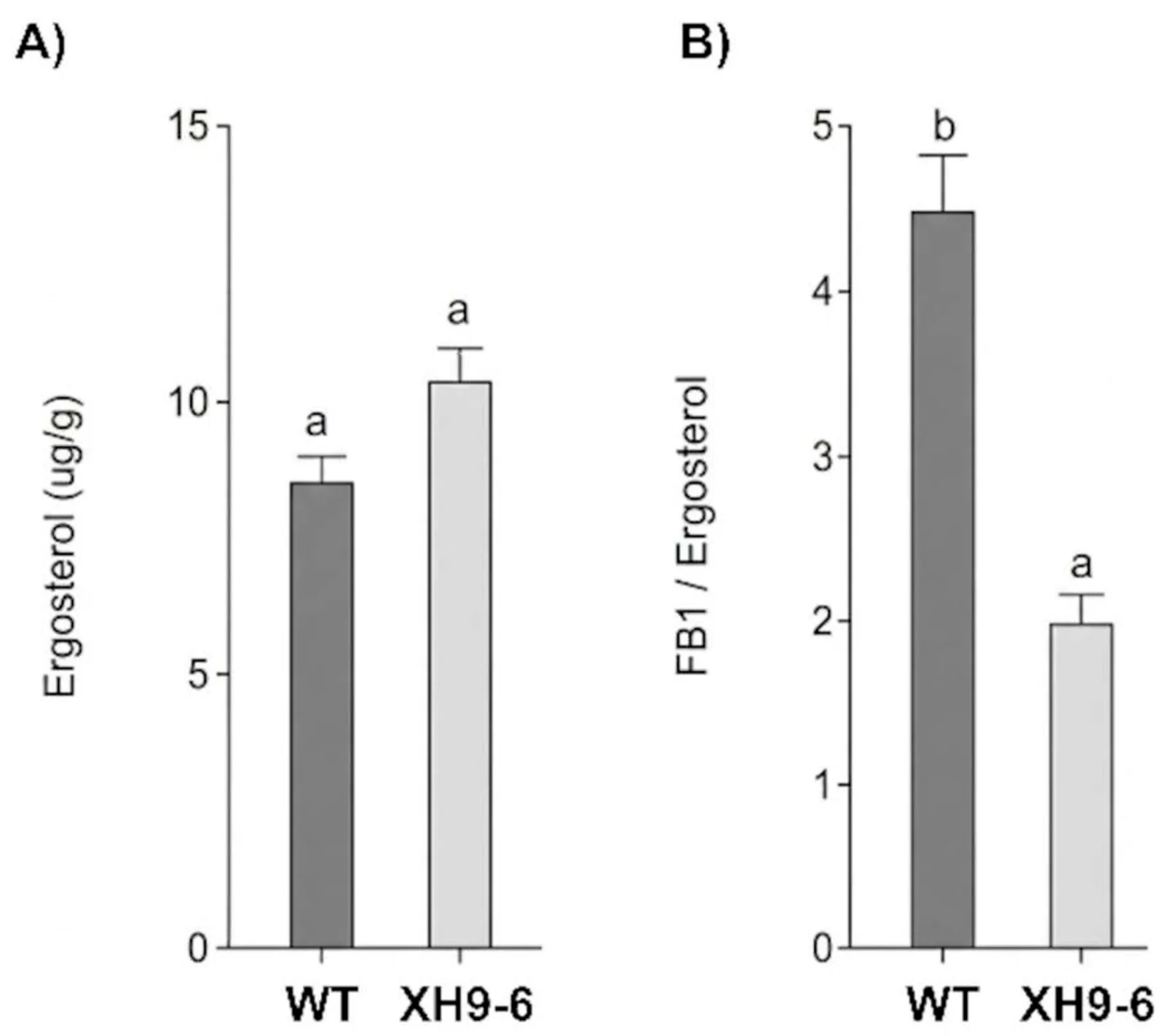
FB1 and ergosterol quantification in maize ears grown under field-like conditions. (**A**) Ergosterol content (µg of ergosterol per g of maize sample) used as a proxy for fungal biomass and ear colonization. (**B**) Ratio of FB1 to ergosterol content (FB1/Ergosterol), indicating specific toxin production normalized by fungal biomass. The samples from all harvested ears per treatment (*n* = 20 per treatment) were pooled to obtain a composite sample, which was analyzed in five subsamples. Bars represent the mean ± standard error (SE). Bars with different letters indicate significant differences according to the DGC multiple comparisons test (*p* < 0.05). nd: means not detectable.

This trend was supported by ergosterol quantification, used as a proxy for fungal biomass, which revealed no significant differences between WT- and XH9-6-infected ears (Figure 4A), and therefore broadly comparable levels of colonization. When normalized to ergosterol content, XH9-6-treated samples displayed the lowest FB1/ERG ratios among all fungal treatments (Figure 4B). These results are consistent with an effect of CHV1 on downregulating FB1 biosynthesis in planta rather than on reducing fungal growth. Taken together, our results demonstrate a clear association between CHV1 infection and reduced FB1 accumulation across two experimental systems. Firstly, CHV1 consistently reduced FB1 production in autoclave-sterilized maize kernels under controlled laboratory conditions, as demonstrated in four independent transformants. Secondly, this reduction was also observed in maize grains harvested from CHV1-infected plants grown under greenhouse and field-like conditions. These findings provide strong evidence that CHV1 acts as a hypomycotoxigenicity-associated factor in *F. verticillioides*.

There were no previous reports of CHV1 affecting FB1 biosynthesis. In *F. graminearum,* CHV1 was shown to have an effect on the production of another mycotoxin, deoxynivalenol (DON) [27]. However, DON levels were increased in the presence of CHV1 rather than reduced, as we observed for FB1 in *F. verticillioides*. Mycotoxins are secondary metabolites produced through species-specific pathways that are tightly regulated by both pathway-specific regulators and global transcriptional networks. Mitogen-activated protein kinase (MAPK) signaling, along with heterotrimeric G protein pathways, has been implicated in the regulation of fumonisin biosynthesis in *F. verticillioides* [48,49]. Notably, CHV1 infection is known to alter G-protein [50,51] and MAPK signaling [52,53] in *C. parasitica*. We hypothesize that the observed reduction in FB1 production may result from interference of CHV1 with these signaling pathways in *F. verticillioides* as it occurs in its natural host fungus. Therefore, future studies evaluating the expression of key genes within the FUM biosynthetic cluster, including the pathway-specific transcription factor FUM21, together with global regulatory factors such as AreA and PacC and MAPK-mediated signaling networks, will be required to test this hypothesis and elucidate the molecular mechanisms underlying CHV1-induced downregulation of FB1 production.

### 3.4. CHV1 Is Not Transmitted Through Hyphal Anastomosis Within the Genetic Background of Fusarium verticillioides Strain M3125

Mycoviruses are transmitted horizontally mainly through hyphal anastomosis. Hence, the parasexual cycle involving exchange of genetic material through fusion of vegetative hyphae is expected to be the main route by which mycovirus-associated phenotypic traits are spread in the host fungus population. After corroborating the ability of CHV1 to efficiently replicate in *F. verticillioides*, inducing a marked reduction in FB1 production, we investigated its transmission by hyphal anastomosis in this heterologous host. For that, we paired CHV1-infected strain XH9-6 with a virus recipient strain tagged with a marker conferring resistance to NTC. The co-cultivation period was extended to 30 days to maximize the likelihood of detecting viral transmission in this heterologous system, following preliminary assessments at 7 and 14 days in which no viral transfer was observed. Unexpectedly, after repeated transmission attempts, none of the NTC-resistant isolates obtained after co-cultivation with XH9-6 showed the presence of CHV1. This result contrasted with previous reports on the successful horizontal transmission of CHV1 in heterologous hosts [24,26,54], including Fusarium species [27,28]. Fungi have developed genetic mechanisms that result in vegetative incompatibility to limit hyphal anastomosis between different strains [55,56]. The process of vegetative compatibility/incompatibility is controlled by allelic differences at a number of gene *loci* that can vary considerably among species [55]. Strains compatible to undergo hyphal anastomosis are placed in the same vegetative compatibility group (VCG). *Fusarium verticilliodes* has a very complex VCG structure with many VCGs composed of only one or two members [57,58,59,60]. Bearing this in mind, in our transmission assays the virus donor and recipient strains were both generated in the same genetic background, that of type strain M3125, and yet no transmission was observed. It should be noted, though, that self-incompatibility, that is, strains that cannot anastomose with themselves, has been described in a variety of fungi, including *F. verticillioides* [61]. Further work will be required to determine if our result regarding failed horizontal transmission of CHV1 in *F. verticillioides* is associated with the specific strain we used, or a general feature of the *F. verticillioides*—CHV1 interaction. It is also worth mentioning that although not studied here, sexual reproduction might function as an avenue for the transmission of CHV1 among genetically distinct fungal strains in *F. verticillioides*. In its natural host, *C. parasitica*, sexual spores do not carry CHV1 [62,63], but in the heterologous host *F. graminearum* [27], efficient transmission of CHV1 through sexual reproduction was reported. These contrasting results suggest that each fungal host—hypovirus interaction might have unique features that have to be investigated.

## 4. Conclusions and Future Perspectives

Certain biological factors must be considered when evaluating the feasibility of control strategies based on the use of heterologous mycoviruses. In particular, successful implementation will depend on (i) the capacity of the mycovirus to consistently reduce virulence and/or toxin production in the target host under relevant environmental conditions, (ii) its ability to replicate and remain stable within the new host, and (iii) its capacity for effective dissemination. In this regard, our findings indicate that CHV1 has features of interest for the biological control of *F. verticillioides* as it significantly reduces FB1 production. We also showed that CHV1 stably infects *F. verticillioides*, efficiently replicating in the cytoplasm of host cells. These results provide evidence that CHV1-infected isolates might have the potential to compete with toxigenic virus-free *F. verticillioides* populations in maize. In contrast, we found no evidence of horizontal transmission of CHV1 in *F. verticillioides* through the usual route of hyphal anastomosis in the genetic background of the strain used in this study, type strain M3125, and in the conditions tested. Considering that efficient horizontal transmission of a mycovirus is very relevant for its potential application in biological control, future work will examine in more detail this aspect of the *F. verticilloides—*CHV1 interaction. For instance, by extending the work to other *F. verticillioides* strains and by analyzing sexual reproduction as another potential route of CHV1 dissemination among genetically distinct fungal strains through transmission via sexual spores. Furthermore, the role of antiviral defense mechanisms such as RNA silencing in modulating the *F. verticillioides*–CHV1 interaction will be investigated.

## Figures and Tables

**Table 1:** Growth rate (mm^2^/day) and conidia production of strains infected with the CHV1 virus and the virus-free wild type.

*F. verticillioides* Strain	Growth Rate (mm^2^/day)	Conidia Production ∙ 10^4^ (Conidia/mm^2^)
WT	646.45 ± 3.94 ^d^	3.3 ± 0.6 ^b^
XH9-6	506.88 ± 8.58 ^b^	4.3 ± 1.0 ^b^
XH9-9	317.56 ± 5.79 ^a^	5.1 ± 0.8 ^b^
XH9-13	587.36 ± 8.25 ^c^	0.8 ± 0.1 ^a^
XH9-25	582.16 ± 7.62 ^c^	3.9 ± 0.6 ^b^

Strains (WT and CHV1-infected) were inoculated onto PDA plates with 5 µL of a 1.0 × 10^6^ conidia/mL suspension and incubated at 25 °C. Colony radius (mm^2^) was measured daily until colonies almost reached the plate edge (6–7 days). Data represent the mean ± standard error (SE). Mean values with different letters within the same column indicate significant differences between strains, according to the DGC multiple comparisons test (*p* < 0.05). Five replicates were prepared per strain, and the experiment was repeated twice.

**Table 2:** Effect of CHV1 on FB1 production by *F. verticillioides* in maize kernels.

*F. verticillioides* Strain	FB1 Production
WT	458.75 ± 97.13 ^b^
XH9-6	217.33 ± 41.44 ^a^
XH9-9	217.09 ± 53.08 ^a^
XH9-13	130.26 ± 23.12 ^a^
XH9-25	276.20 ± 68.68 ^a^

Sterile maize kernels placed in Erlenmeyer flasks were inoculated with 1 mL of a conidial suspension (1.0 × 10^6^ conidia/mL) of virus-free wild type or CHV1-infected, and incubated in the dark at 25 °C for 28 days. Data are expressed as µg of FB1 per gram of maize (µg/g). Data are represented as means ± SE. Mean values with different letters within the same column indicate significant differences between strains, according to the DGC multiple comparisons test (*p* < 0.05). Six replicates were prepared per strain, and the experiment was repeated twice.

## Data Availability

All data supporting this study are available within the article.

## Supplementary Materials

The following supporting information can be downloaded at: https://www.mdpi.com/article/10.3390/v18090971/s1, Figure S1: Transformation of *Fusarium verticillioides* with the *Cryphonectria hypovirus* 1 (CHV1) infectious cDNA clone. Table S1. Primers used in this study.
