# Supplementary material for: Heterologous Expression of Cryphonectria Hypovirus 1 in Fusarium verticillioides Reveals Stable Replication and Reduced Fumonisin Production
Source: Viruses. 2026 Sep 3;18(9):971. doi: 10.3390/v18090971 (PMC13611953; doi:10.3390/v18090971)
Supplement: Supplementary file 1 [file viruses-18-00971-s001.zip › Supplementary Materials.pdf]

Supplementary Materials

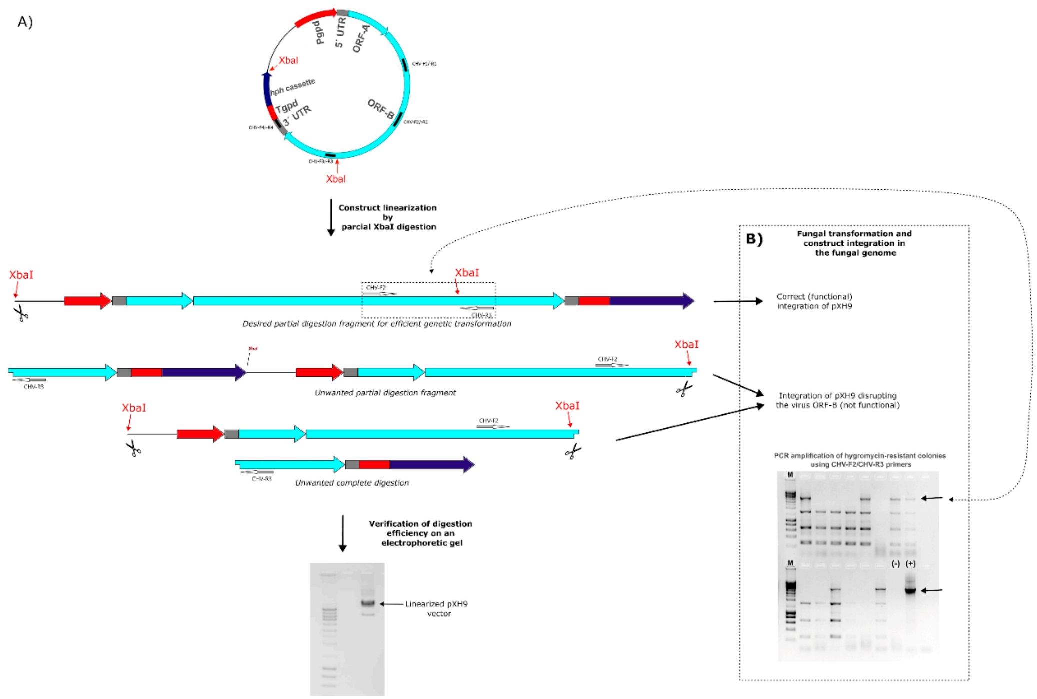

Figure S1. Transformation of *Fusarium verticillioides* with the *Cryphonectria hypovirus* 1 (CHV1) infectious cDNA clone. (A) Schematic representation of pXH9 linearization, fungal transformation, and construct integration into the fungal genome. The CHV1 infectious cDNA clone pXH9 (Chen *et al.*, 1993) contains a full-length cDNA copy of the CHV1 genome, including the 5′ and 3′ untranslated regions (UTRs) and ORF-A and ORF-B, under the control of the *Cryphonectria parasitica* glyceraldehyde-3-phosphate dehydrogenase promoter (Pgpd) and terminator (Tgpd). The plasmid also carries a hygromycin B phosphotransferase (*hph*) cassette for selection of hygromycin-resistant transformants. To promote genomic integration, pXH9 was subjected to partial digestion with XbaI. Because the plasmid contains two XbaI restriction sites, digestion was performed for 10 min at 37 °C, generating predominantly partially linearized molecules. Only transformants derived from plasmids linearized without interruption of the CHV1 ORF-B are expected to retain a functional viral genome following integration. Black boxes indicate primer binding sites (CHV-F/R series), and red arrows denote XbaI restriction sites. **(B)** PCR screening of hygromycin-resistant transformants using primers CHV-F2 and CHV-R3. M, 1 kb DNA ladder (NZYDNA Ladder III, NZYTech®); lanes 1–14, independent hygromycin-resistant transformants; (−), no-template control; (+),

positive control (pXH9 plasmid DNA). The arrow indicates the expected 3,980-bp amplicon produced when pXH9 is integrated without disruption of CHV1 ORF-B. Additional nonspecific amplification products were occasionally detected, likely resulting from suboptimal PCR conditions and/or secondary structures within the viral cDNA template. These bands did not interfere with the identification of the expected diagnostic amplicon. DNA fragments were stained with ethidium bromide (approximately 0.25 μg mL^{-1}) and visualized under UV illumination using an EC3 Imaging System (UVP, LLC).

Table S1. Primers used in this study

**Primer name**	**Sequence (5′ → 3′)**
CHV1-F1	CAGTCGCTGATTATGTGAAAGCG
CHV1-R1	CAAGGAACGAGGATGTCAAAACC
CHV1-F2	AAGTACTTGGTTGGCATGGCGT
CHV1-R2	ATACAGCCTTGATGTGTCCCAC
CHV1-F3	GATCGCTTTATCGCCAAACTGG
CHV1-R3	GAATAAACAGCTCGACCCAATGC
CHV1-F4	CAAAGGAGCAGGAAACACAGTG
CHV1-R4	CGATCCCTATACCCTTTGTTGTC
